# Considerations of the Genetic Background of Obesity among Patients with Psoriasis

**DOI:** 10.3390/genes14030594

**Published:** 2023-02-26

**Authors:** Anna Czarnecka, Dorota Purzycka-Bohdan, Monika Zabłotna, Michał Bohdan, Roman J. Nowicki, Aneta Szczerkowska-Dobosz

**Affiliations:** 1 Department of Dermatology, Venereology and Allergology, Medical University of Gdansk, 80-210 Gdansk, Poland; 2 First Department of Cardiology, Medical University of Gdansk, 80-210 Gdansk, Poland

**Keywords:** psoriasis, obesity, genetic background

## Abstract

Psoriasis comorbidities may emerge from pleiotropic mechanisms, including common proinflammatory pathways, cellular mediators or genetic predisposition. Obesity is considered to be an independent risk factor of psoriasis, which may influence the severity of the disease and its early onset, decrease patients’ quality of life, alter response to psoriasis therapies and affect morbidity by reduced life expectancy due to cardiovascular events. Although novel approaches, including genetic techniques, have provided a wide range of new research, there are still scarce studies elaborating on the common genetic background of psoriasis and obesity. The aim of this study was to present and evaluate a possible common genetic background of psoriasis and concomitant increased body mass based on the review of the available literature.

## 1. Introduction

Psoriasis is a common, chronic, immune-mediated inflammatory skin disease characterized by the formation of erythematous scaling plaques. Psoriasis is regarded as a complex disorder of multifactorial origin. The combination of immunological, genetic and environmental factors (mainly emotional stress, smoking habits, lifestyle, diet, physical activity and infections) plays a key role in its etiology [1]. The primary linkage analysis of genetic studies among psoriatic patients of familial incidence revealed multiple *PSORS* loci related to psoriasis [2]. The *PSORS1* loci (psoriasis susceptibility 1 locus) within the major histocompatibility complex region (MHC) on the chromosome 6p21.3 was identified as responsible for about 30–50% of psoriasis heritability. Major early-onset psoriasis (before 40 years of age) susceptibility was linked to the HLA-Cw6*06 gene, which is associated with human leukocyte antigen (HLA) [3]. Furthermore, the immune system plays a crucial function in the formation of psoriatic skin lesions. This involves the production of numerous proinflammatory cytokines, such as tumor necrosis factor alpha (TNF-α), interferon alpha (INF-α) and interleukin 6 (Il-6), as well as activation of the Th1 and Th17 lymphocytes, which leads to cascade production of the main psoriasis mediators—mainly interleukin 17 (Il-17), interleukin 12 (Il-12) and interleukin 23 (Il-23) [4,5]. In light of the current understanding of psoriasis pathogenesis, the disease is recognized as a systemic disorder due to associated chronic systemic inflammation. There is evidence that it subsequently leads to the development of a wide spectrum of disorders comorbid with psoriasis, such as obesity, hypertension, diabetes, metabolic syndrome and cardiovascular disease (CVD) [6,7].

Obesity seems to be a crucial triggering factor for the evolution of psoriasis comorbidities [8], which may evolve not only from common inflammatory pathways, but also due to a shared genetic background. This hypothesis emerged from studies on same-gender siblings as genetic controls, which showed an association of body mass index (BMI) with psoriasis [9]. Further research confirmed this observation in a large cross-sectional study on Danish twins, which implied a common genetic etiology for psoriasis and obesity [10]. Highly multifactorial genetic heritability of body weight and obesity accounts for approximately 65–80% of its occurrence [11]. Nevertheless, the link between obesity and psoriasis remains unclear, and may arise from shared genetic polymorphisms, aberrant regulation of proinflammatory and metabolic molecular trails or environmental factors, mainly lifestyle, for instance: a high-calorie diet, reduced physical activity, smoking, alcohol consumption or emotional stress [12,13].

The multifactorial nature of psoriasis and its association with chronic systemic inflammation highlights the complexity and significance of the disease and the need for ongoing research to better understand its pathogenesis and concomitant comorbidities. Therefore, this study aimed to shed light on the possible shared genetic basis between psoriasis and obesity. In order to accomplish this goal, the authors conducted a comprehensive review of the existing literature by searching the PubMed database using relevant keywords, such as “psoriasis”, “obesity”, “BMI”, “genetic” and “polymorphism”. The publication aimed to present the current knowledge and to provide insights into the potential common genetic background between these two complex disorders, as well as the potential role of obesity in the development and severity of psoriasis.

## 2. Epidemiology of Overweight and Obesity among Psoriatic Patients and Its Clinical Consequences

Epidemiological reports noted a variable prevalence of obesity among psoriasis patients, reaching from 15 to 30%; however, most studies recruited individuals only of Caucasian ancestry [14]. There is evidence that the overabundance of adipose tissue via chronic systemic inflammation may increase both the psoriasis incidence risk and its severity, which also correlates with body mass index values [15,16]. The cross-sectional PSO HEALTH 3 study evaluated the overweight and obesity prevalence among 1265 psoriatic patients in Germany. The results confirmed significantly higher BMI values in patients with psoriasis in comparison to general population (mean BMI 28.0 kg/m^2^ and 25.9 kg/m^2^, respectively) [17]. Furthermore, the HUNT prospective study in the Norwegian population assessed the risk of psoriasis incidence in relation to BMI, waist circumference, waist-to-hip ratio and 10-year body weight alteration. In general, a twofold increase in psoriasis risk was observed among individuals with obesity and high abdominal mass [18]. A nationwide population-based prospective cohort study on 399,461 psoriasis patients of Korean origin confirmed the relationship between abdominal obesity and the disease’s prevalence [19].

There is strong evidence that psoriasis comorbidities may emerge from pleiotropic mechanisms including common proinflammatory pathways, cellular mediators and genetic predisposition. To date, robust research has enabled the identification of a vast range of psoriasis comorbidities, such as cardiometabolic diseases (hypertension, diabetes, hyperlipidemia, metabolic syndrome), gastrointestinal diseases (inflammatory bowel disease, non-alcoholic fatty liver disease), chronic kidney disease, malignancy, infection and mood disorders (depression, anxiety and suicidal thoughts). Nonetheless, obesity is considered an independent risk factor of psoriasis [20] which may influence the severity of the disease and its early onset, decrease patients’ life quality, alter the response to psoriasis therapies and affect morbidity by a reduced life expectancy due to cardiovascular events [21,22,23]. For instance, the study conducted by Horreau et al. aimed to assess the incidence of cardiovascular events (CVE) in patients with psoriasis and psoriatic arthritis through a meta-analysis of 33 observational studies. The results indicated that patients with psoriasis and psoriatic arthritis have an elevated risk of developing a myocardial infarction (MI) compared to the general population. The calculated odds ratio for myocardial infarction in patients with psoriasis was 1.25 (95% confidence interval [CI] 1.03–1.52) and in patients with psoriatic arthritis, it was 1.57 (95% CI 1.08–2.27). The findings further revealed that the risk of myocardial infarction was more pronounced in patients with severe psoriasis and early-onset psoriasis [24].

## 3. Association of Anthropometric and Body Composition Measurements with Psoriasis

Anthropometric and body composition measurements play an important role in understanding the association between obesity and psoriasis. The relationship between body composition measurements and psoriasis is complex and multifactorial, with the interplay between genetic factors, lifestyle, diet and environmental factors contributing to the development of obesity among psoriatic patients. Although BMI is the most common tool for overweight and obesity evaluation, it fails to determine the constituents of the body mass composition, such as fat mass (adipose tissue), fat-free mass and water distribution (extracellular). The proportions of these factors constitute a practical indicator of metabolic health that can be used as a predictor of cardiometabolic risk, especially in the context of psoriasis patients [25]. Apart from body mass index, other anthropometric measurements, such as waist circumference, waist-to-hip ratio and weight alteration over a period of 10 years, have also been linked to the incidence of psoriasis, as in the aforementioned HUNT study [18]. Recently, bioelectrical impedance analysis (BIA) has been adapted as non-invasive, low-cost, time-saving and precise technique for body mass composition assessment. The method involves an analysis of the weak electric current flow through the body tissues, which differ in resistance and reactance measurement. The discrepancies in the tissues’ impedance signals are analyzed to calculate the proportions of fatty tissue, muscle mass and water distribution. It is crucial to highlight that the results obtained from bioelectrical impedance analyses are not free of limitations and should be interpreted with care, as the accuracy of BIA may be impacted by various factors, such as an individual’s hydration status, body temperature and age [26,27,28].

There are scarce studies considering the relationship between psoriasis and body mass composition. The systematic review by Blake et al. provided evidence for an association between increased overall body fat and visceral fat among psoriatic patients in comparison to controls [29]. Furthermore, Galluzzo et al. analyzed bioelectrical impedance parameters among 164 adult patients with psoriasis and found an association with high fat mass values (53.3% of women and 48.1% of men were obese). Furthermore, the BIA method was found to be more precise in obesity screening compared to the BMI approach [30]. Additionally, BIA has been used in combination with other tools, such as skinfold thickness measurement, dual-energy X-ray absorptiometry (DXA) and magnetic resonance imaging (MRI), to provide a more comprehensive evaluation of body composition in psoriasis patients [31].

In conclusion, a more comprehensive evaluation of the correlations between anthropometric and body mass composition parameters and psoriasis could provide significant implications for the treatment and management of the disease in the future. Such insights could facilitate the design of more effective psoriasis therapeutic strategies, reducing both cardiovascular risk and the overall burden of psoriasis-associated morbidity and improving quality of life.

## 4. Molecular Mechanisms of Obesity among Psoriatic Patients

Adipose tissue is responsible for energy storage and lipid synthesis; however, currently, it is considered as an endocrine organ involved in active adipocyte synthesis of adipokines, hormones and a wide range of proinflammatory cytokines (primarily IL-6 and TNF-a). Due to the recently discovered association of adipose tissue (primarily the visceral type) with variety of molecular pathways, it is now acknowledged as element of the innate immune system [32]. The psoriasis-driven chronic systemic inflammation and resulting cascade of metabolic comorbidities (obesity, insulin resistance, dyslipidemia, hypertension, endothelial damage, atherosclerosis and premature cardiovascular disease) comprise a so-called “psoriasis-march” concept [33].

Recent studies have shown that the functions and levels of circulating adipokines play a crucial role in linking the pathophysiology of psoriasis with obesity [34]. Adiponectin acts as an anti-inflammatory protein with protective function over insulin resistance and endothelial fatty acid accumulation. Many studies have confirmed reduced adiponectin levels in both psoriasis and obese individuals [35,36]. Leptin is responsible for satiety regulation and systemic energy homeostasis. The analysis of plasma leptin concentrations showed increased values among both obese and psoriatic subjects, and, furthermore, these values were positively correlated with BMI and PASI levels. Moreover, newly identified adipokines, such as resisitin, chemerin, visfatin, omentin and vaspin, also play a role in obesity prevalence among psoriatic patients; however, more research is needed to elucidate their exact role [37]. Apart from adipokines, cytokines may also influence common molecular mechanisms of obesity and psoriasis. Sumarac-Dumanovic et al. found overstimulation of the Il-23/Il-27 axis among obese women, which also plays a major role in psoriasis pathogenesis and psoriasis-linked comorbidities [38,39].

Furthermore, recent studies have emphasized the contribution of epigenetic mechanisms to both psoriasis and obesity. Genetic alterations triggered by DNA methylation, histone modifications and the influence of non-coding RNAs, alongside environmental factors and lifestyle changes, may be key components of body mass changes in patients with psoriasis [40,41].

## 5. Genome Wide Association Studies (GWAS) Studies on Psoriasis and Obesity

Genome-wide association studies (GWAS) have emerged as a powerful tool for identifying genetic variants (chiefly single-nucleotide polymorphisms) that contribute to the pathogenesis of common complex diseases, such as psoriasis. The study technique employs high-throughput genotyping technology to scan the entire genome of large case-control datasets for genetic markers that are associated with a trait. Additionally, GWAS studies uncovered the contribution of genetic variations in non-HLA loci to the pathogenesis of psoriasis. These findings have broadened the understanding of the underlying biological processes involved in psoriasis development, including cytokine signaling, T cell activation and inflammation [42].

The new era of genome wide association studies enabled further identification of over 60 single-nucleotide polymorphisms (SNPs) involved in the genetic background of psoriasis among Caucasian and Asian populations [43]. The majority of them involve transcripts and non-coding regions near genes encoding for IL-23/Th17 signaling *(Il23R*, *IL23A*, *IL12B*), skin barrier function (*LCE3B*, *LCE3C*) and IFN and NF-κB signaling, as well as innate immunity (*TNFAIP3*, *TNIP1*, *NFKBIA*, *REL*, *TYK2*, *UBE2L3*, *CARD14*, *CARD6*, *IFIH1*), adaptive immunity (*ERAP1*, *ZAP70*) and Th-2 activation response (*Il-4*, *Il-13*) [1,44,45,46]. However, to date, genetic investigation of the genetic background of psoriasis managed to explain less than 30% of its traits, which may suggest the involvement of other mechanisms such as gene-to-gene and gene–environmental interactions or epigenetic changes [47].

Although GWAS provides a novel approach to the genetic research, there are still a limited number of studies elaborating on the common genetic background of psoriasis and obesity. The Mendelian randomization study by Budu-Aggrey et al. evaluated the causal relationship between higher BMI values and greater psoriasis incidence. A large-population database and published genome-wide-association studies were employed for the analysis. Additionally, the investigation of the potential pleiotropic effects of BMI-associated single-nucleotide polymorphisms showed comparable results with the isolated effect of the *FTO* gene variant, which emphasizes its important role in obesity [48]. On the other hand, the first genome wide association study among a Polish psoriatic population compared the genetic backgrounds related to obesity among psoriatic patients and healthy controls by means of GWAS. The results showed significant interactions between 11 SNPs and abnormal body mass indices among psoriatic patients. One particular variant of the *FTO* gene, precisely the A allele of rs1558902, was found to be significantly associated with higher body mass index values, predominantly in individuals with type I psoriasis. Additionally, four alternative alleles (such as rs1556519 in the *ITLN2* gene, rs12972098 in the *AC003006.7* gene, rs12676670 in the *PAG1* gene and rs1321529) were determined to be associated with a reduced BMI among psoriatic patients [49].

To date, with the help of GWAS approach analysis, over 870 obesity-related SNPs were identified. They determine merely 3–5% of the “common”/multifactorial obesity variability, which still remains to be determined [50]. Highly infrequent single gene mutations (such as *LEP*, *LEPR* and *POMC*) stand for rare cases of monogenic obesity; however, some of them are also related to polygenic obesity (such as *MC4R* and *FTO*) [51]. GWAS studies identified multiple common obesity-susceptible loci (*FTO*, *MC4R*, *MC3R*, *SLC6A14*, *PCSK1*, *TMEM18*, *POMC*, *BDNF* and *NEGR1)* [52]. They take part in the regulation of body mass system mechanisms, including the neurological trail of appetite and satiety, insulin secretion, adipogenesis, energy and lipid metabolism and gene–environment interactions (diet and physical activity) [51]. Moreover, recent findings have suggested an overlap of obesity-related single-nucleotide polymorphisms with associated other metabolic diseases (mainly hypertension, type 2 diabetes and cardiovascular disease) [51].

The use of genome-wide association studies proved to be important in advancing our understanding of both psoriasis and obesity, as well as in identifying the genetic variations associated with these complex diseases. However, while progress has been made in understanding the genetic basis of obesity and psoriasis, much remains to be discovered. In the future, it may provide important insights into the development of new strategies for obesity prevention and treatment among psoriatic patients.

## 6. Potential Common Genetic Background of Obesity among Psoriatic Patients

### 6.1. Gene Polymorphisms Associated with Obesity

To date, the *FTO* gene (localized on chromosome 16) has been identified to have the greatest impact on the obesity trait in GWAS studies [53], and in addition, showed association with BMI value, hip circumference and body weight [54]. It codes for 2-oxoglutarate-dependent nucleic acid demethylase, a protein involved in the regulation of food intake and body energy expenditure, which results in body fat mass control. The highest *FTO* mRNA expression was found in hypothalamic nuclei, an energy homeostasis regulating center [55]. The study by Coto-Segura et al. showed an association between the *FTO* gene polymorphism (rs9930506) and increased mean BMI scores among psoriatic patients of Spanish origin (European population). The correlation translates into elevated obesity risk, especially in individuals who are homozygous for the SNP’s risk allele. On the other hand, the authors did not find any impact of the risk allele on the disease’s severity [56]. Similar results among Polish psoriatic patients were described by Tupikowska-Marzec et al. The *FTO* gene rs9939609 variant corresponded with higher BMI values as well as augmented WHR measurement (waist-to-hip ratio), PASI values (Psoriasis Area Severity Index) and serum insulin concentrations among the patient group. However, it is worth to mention that no differences in the risk allele prevalence were found in the study group [57].

The meta-analysis of GWAS results among large population of European origin proved an association between common genetic variants in proximity to *MC4R* gene and fat mass, body weight and early-onset severe obesity. The *MC4R* gene is localized on chromosome 18q21 and codes hypothalamic melanocortin 4 receptor [58]. To date, there is little research analyzing the *MC4R* gene’s influence on psoriasis patients. A cross-sectional study among psoriatic patients of Romanian descent focusing on *MC4R* gene polymorphisms showed a significantly increased risk of obesity, diabetes mellitus and psoriatic arthritis. At the same time, the authors did not find any association between *TNF* gene polymorphisms and body composition among psoriatic patients. Still, further evaluation of the results on a large-scale population is required [59].

### 6.2. Gene Polymorphisms Associated with Lipid Metabolism

The evaluation of genetic association between the adiponectin gene (*ADIPOQ*) and leptin and leptin receptor genes (*LEP*, *LEPR*) was ambiguous. Some authors did not observe any genetic links between those polymorphisms and adipose tissue among psoriatic patients [60,61]. On the other hand, the *ADIPOQ* and *LEP* genes were found to play a notable role in the gene-to-gene interaction networks in the genomes of psoriasis patients [62]. Functional analysis of the *LEP* gene polymorphism (G-2548A) by Abdel Hey et al. concluded that it could serve as both a prognosticator of metabolic syndrome among psoriasis subjects and the skin disorder risk itself [63]. Moreover, a population-based case control study among 574 psoriasis subjects of Hungarian origin demonstrated the link between the *LEPR* gene polymorphism (rs1137101) and obesity prevalence among patients with early disease onset. Multiple single-nucleotide polymorphisms were genotyped in the study, such as *ADIPOQ*, *FTO*, *PPARG* and *FTO*; however, no prominent associations were found [64]. Mitsuyama et al. described the significant association between leptin mRNA expression in subcutaneous adipose tissue among psoriatic patients and serum leptin levels, disease severity and obesity [65]. Hence, the link between the *ADIPOQ* gene, the *LEP* gene and the *LEPR* gene polymorphisms and adipose tissue in psoriatic patients is inconclusive and requires further evaluation in large prospetiveprospective studies.

Functional analysis of the peroxisome proliferator-activated receptor gamma gene (*PPARG*) revealed its regulating role in adipocyte maturation and the insulin-signaling pathway, which indirectly influences metabolic aberrations [66]. To date, little is known about its role as a psoriasis susceptibility factor; however, in in vivo studies on psoriasis models in mice skin, PPAR-*γ* levels were decreased, which may activate the proinflammatory response of the immune system [67]. Seleit et al. discovered a positive correlation between the Pro12Ala polymorphism of the *PPAR-γ* gene and obesity among psoriatic patients, although the results need to be confirmed in further large prospective studies [68]. Apart from that, observations show that orally administered pioglitazone, which acts as a PPAR-*γ* agonist, alleviated the skin inflammation among obese psoriatic patients [69]. The finding mentioned above provides evidence that PPAR-*γ* plays a significant role in modulating adipocyte maturation and insulin signaling. For this reason, it has been the subject of investigation in the context of psoriasis, demonstrating potential associations with obesity and the manifestation of skin inflammation.

Recent data imply that *PCSK9* gene polymorphisms might be another possible link between psoriasis and obesity. They code for proprotein convertase subtilisin/kexin type 9 (PCSK9), which promotes degradation of the low-density lipoprotein receptor (LDLR) and, thus, elevate serum LDL levels [70]. There is growing evidence that *PCSK9* gene mutations are directly interrelated with body mass index (BMI), hypercholesterolemia and coronary artery disease (CAD) [71,72]. Furthermore, PCSK9, in the past few years, has also been recognized as a potential psoriasis trigger and co-factor promoting psoriasis comorbidities [73]. The analysis of the PCSK9 mRNA expression in the psoriatic lesions proved a significant difference (5 times) in comparison to healthy skin levels [74]. Merleev et al. showed that *PCSK9* SNPs might serve as psoriasis susceptibility loci. By means of RNA-Seq-based variant detection, the authors uncovered a potential psoriasis susceptibility locus at 1p32.3 within the gene *PCSK9* (rs662145). Consequently, the hypothesis was confirmed through the analysis of independent genomic and RNA-Seq datasets. Single-cell RNA-Seq analysis identified keratinocytes as the primary source of PCSK9 in human skin. However, the expression of PCSK9 was not homogeneous across all keratinocyte subpopulations. Further analysis through single-cell RNA-Seq and immunohistochemistry revealed an epidermal gradient of PCSK9 expression, with the highest expression observed in basal and early spinous layer keratinocytes and the lowest expression found in granular layer keratinocytes [75]. Another case control study determined *PCKS9* polymorphisms to be connected with obesity risk and CAD in the Chinese Han population. The authors revealed the link between the rs2483205 polymorphism of the *PCSK9* gene and an elevated risk of obesity. The presence of the G allele at rs562556, on the other hand, was associated with decreased levels of low-density lipoprotein cholesterol (LDL-C), blood glucose, body mass index (BMI) and mean platelet volume (MPV), which may further lead to a greater risk of cardiovascular events. These associations were statistically significant, with *p*-values of less than 0.05 [76]. To conclude, *PCSK9* gene polymorphisms might be a promising trait to evaluate in future genetic studies of obesity among psoriatic patients.

### 6.3. Gene Polymorphisms Associated with Psoriasis

The major psoriasis susceptibility locus HLA-Cw*06 showed a significant association with BMI, waist-to-hip-ratio (WHR), and obesity among psoriatic patients of Chinese Han descent. A case control study by Jin et al. aimed to investigate the associations between genetic susceptibility, body weight and waist-to-hip ratio and the risk of developing psoriasis. The results showed that individuals with the HLA-Cw*06 allele had an 8.33-fold increase in the risk of psoriasis compared to those with the HLA-*Cw6* allele. This risk was further exacerbated in individuals with HLA-Cw*06 and a high body weight, where the risk increased to 35 times that of individuals with HLA-Cw*06 and a non-overweight status. Similarly, individuals with HLA-Cw*06 and a high waist-to-hip ratio had a 17-fold increase in risk compared to those with HLA-Cw*06 and a low waist-to-hip ratio. These results suggest that the combined effect of genetic susceptibility, body weight and waist-to-hip ratio contribute to the development of psoriasis [77]. Thus, this study underscores the importance of considering both genetic and lifestyle factors in the assessment of psoriasis risk. The results highlight the significance of identifying individuals with the HLA-Cw*06 allele and high body weight or waist-to-hip ratio, as they may be at a higher risk of developing psoriasis. This information may be utilized in the implementation of personalized prevention and management strategies for individuals at risk.

Finally, a post-GWAS analysis of the psoriasis-promoting cytokines by Li et al. demonstrated elevated disease prevalence among obese patients carrying an *IL12B* gene polymorphism (rs 3212227). Moreover, the association was modified by the levels of visceral and overall adiposity due to the polymorphism interactions with waist circumference and waist-to-hip ratio [78]. The *IL12B* gene encodes the IL12-p40 subunit, which constitutes a critical component of both the IL-12 and IL-23 cytokines. Studies have revealed that these cytokines induce the proliferation of Th1 cells that produce interferon-γ and Th17 cells, which triggers the IL-17 response. These findings implicate the *IL12B* gene in the pathogenesis of autoimmune inflammatory disorders such as psoriasis [79]. On the other hand, the authors of the study did not find any association between the overabundance of fatty mass and the *IL23R* gene (rs7530511) [78].

## 7. Conclusions

Obesity is a complex clinical entity, especially among psoriatic patients, with multiple contributing factors that lead to its development and progression. These factors range from genetic predispositions to lifestyle habits and environmental influences. Multiple studies have provided evidence that a possible common genetic background between obesity and psoriasis may exist due to the interplay of genetic variants that are associated with excessive adipose tissue, altered lipid metabolism and psoriasis susceptibility. This information highlights the need for a more in-depth understanding of the relationship between psoriasis and its comorbidities, especially in the context of obesity.

It is crucial to note that while the genetic component of obesity cannot be modified, other factors, such as lifestyle habits and environmental factors, can be influenced to reduce the risk of obesity in psoriatic patients. For instance, maintaining a healthy diet, engaging in regular physical activity and avoiding exposure to environmental psoriasis triggers may all help to prevent the progression of obesity. Moreover, implementation of these strategies can lessen the severity of the disease and reduce the risk of cardiovascular events, ultimately leading to a better quality of life.

In addition, a better understanding of the interplay between psoriasis and obesity may result in the development of more effective treatment strategies for both conditions. For instance, current therapies for psoriasis focus on reducing systemic inflammation, which is a contributing factor to the progression of obesity. In conclusion, understanding the complex interplay between psoriasis, obesity and its comorbidities is essential for the development of targeted and effective therapies, which will eventually result in improved quality of life for psoriatic patients.

## Data Availability

Not applicable.
